# Muse cells: ushering in a new era of stem cell-based therapy for stroke

**DOI:** 10.1186/s13287-022-03126-1

**Published:** 2022-08-19

**Authors:** Han Li, Jinghui Wei, Xuejia Liu, Ping Zhang, Juntang Lin

**Affiliations:** 1 College of Life Science and Technology, Xinxiang Medical University, Xinxiang, 453003 China; 2 Stem Cell and Biotherapy Engineering Research Center of Henan, Xinxiang Medical University, Xinxiang, 453003 China; 3 College of Biomedical Engineering, Xinxiang Medical University, Xinxiang, 453003 China; 4 Department of Neurology, The First Affiliated Hospital of Xinxiang Medical University, Xinxiang, 45003 China

**Keywords:** Stem cell, Multilineage differentiating stress-enduring cells, Stroke, iPSCs, MSCs

## Abstract

Stem cell-based regenerative therapies have recently become promising and advanced for treating stroke. Mesenchymal stem cells (MSCs) and induced pluripotent stem cells (iPSCs) have received the most attention for treating stroke because of the outstanding paracrine function of MSCs and the three-germ-layer differentiation ability of iPSCs. However, the unsatisfactory homing ability, differentiation, integration, and survival time in vivo limit the effectiveness of MSCs in regenerative medicine. The inherent tumorigenic property of iPSCs renders complete differentiation necessary before transplantation, which is complicated and expensive and affects the consistency among cell batches. Multilineage differentiating stress-enduring (Muse) cells are natural pluripotent stem cells in the connective tissues of nearly every organ and thus are considered nontumorigenic. A single Muse cell can differentiate into all three-germ-layer, preferentially migrate to damaged sites after transplantation, survive in hostile environments, and spontaneously differentiate into tissue-compatible cells, all of which can compensate for the shortcomings of MSCs and iPSCs. This review summarizes the recent progress in understanding the biological properties of Muse cells and highlights the differences between Muse cells and other types of stem cells. Finally, we summarized the current research progress on the application of Muse cells on stroke and challenges from bench to bedside.

## Introduction

Stroke is a cerebrovascular disease caused by the sudden rupture of cerebral blood vessels or arterial occlusion, and can be ischaemic or haemorrhagic [1]. Ischaemic stroke is the most common type, accounting for 80% of all cases [2]. Stroke is the leading and second leading cause of death in China and worldwide, respectively [2], and is characterized by high incidence, mortality, disability, occurrence rates and medical expenses [2]. At present, tissue-type plasminogen activator (TPA) is the only FDA-approved drug for the treatment of acute ischaemic stroke [3]. However, only 3–5% of patients receive TPA due to the narrow time window, contraindications, and risk of complications [2, 4]. Although Jin et al. suggested that stroke-induced compensatory neurogenesis may occur in the hippocampal dentate gyrus and subventricular zone of the rat and human brain, this endogenous neurogenesis is insufficient to achieve functional recovery and reconstruction of neural circuits [5, 6]. In the drug treatment of stroke, neuroprotective agents targeting excitotoxicity, oxidative and nitrifying stress, inflammation and other injury mechanisms have been tested in thousands of nonclinical studies and hundreds of clinical trials [7]. However, a large number of neuroprotective agents fail to translate into clinical use, highlighting the need for the research and development of effective therapeutic methods for stroke [7].

Dozens of clinical studies using neural stem/progenitor cells (NSCs/NPCs) [8–11], mesenchymal stem cells (MSCs) [12–15], and bone marrow mononuclear cells (BM-MNCs) [16–19] have been conducted in the past 10 years due to their tissue regeneration, neurotrophic function and immunomodulatory properties. Although some therapeutic effects have been observed in patients, these effects are not satisfactory or long-lasting. The widespread application of NSCs/NPCs is restricted by their limited sources and ethical issues [9]. MSCs and BM-MNCs rarely differentiate into tissue-specific cells in the host brain and cannot facilitate neural circuit reconstruction [14, 18].

Multilineage differentiating stress-enduring (Muse) cells are adult stem cells first reported in 2010 [20]. In contrast to multipotent MSCs, Muse cells are pluripotent. Accumulating evidences have suggested that Muse cells can migrate to damaged sites, withstand harsh environments, survive for a relative long time, spontaneously differentiate into cells compatible with the homing tissue, and facilitate neural circuit reconstruction [20, 21]. There are numerous review papers describing the application of MSCs, induced pluripotent stem cells (iPSCs) or NSCs for central nervous system disorders, including stroke [4, 22–24]. However, few review papers have covered the application of Muse cells for stroke. This review details the unique molecular and cellular properties of Muse cells and highlights their potential of clinical application on stroke and their possible mechanisms of action.

## Properties of Muse cells

### Cell sources

Muse cells are adult stem cells that were discovered accidentally by Japanese scientist Mari Dezawa in 2007 [25] and first reported in 2010 [20]. Muse cells were identified as cells expressing pluripotent marker stage-specific embryonic antigen 3 (SSEA-3) and MSCs marker CD105. These cells reside in numerous adult tissues, including the bone marrow [20, 26], peripheral blood [27], umbilical cord [28], adipose tissue [29, 30], and dermal fibroblasts [26]. Muse cells are widespread in the connective tissues of each organ because of the adjacent vessels. Given that Muse cells in the peripheral blood enter tissues from the circulatory system, they naturally localize to the connective tissue first. Muse cells comprise approximately 0.01–0.03% of BM mononucleated cells [20, 31], 0.01–0.2% of peripheral blood cells [27], and 1–6% of various MSCs [21, 31]. To date, bone marrow-derived MSCs (BM-MSCs) and umbilical cord-derived MSCs (UC-MSCs) are the most commonly used sources of Muse cells [20, 26]. Recently, we successfully isolated and characterized Muse cells from menstrual blood-derived endometrial stem cells (MenSCs) providing another source for Muse cells (data not published yet). Since Muse cells naturally exist as endogenous cells rather than immortalized or monoclonal amplified tumorigenic cells, the homogeneity of Muse cells is still unknown.

### Safety

Unlike iPSCs, Muse cells naturally exist as stem cells in organisms without artificial induction or manipulation and thus are considered nontumorigenic [32]. The gene expression level of tumorigenic factors in Muse cells is much lower than that in embryonic stem cells (ESCs) and iPSCs [33]. Besides, the levels and pattern of tumorigenic factors in Muse cells are similar to those observed in non-Muse fibroblasts [33]. The length of telomeres and the activity of telomerase are consistent with the replication, pluripotency, and tumorigenicity of stem cells [30, 34]. Wakao et al. compared telomerase activity among fibroblasts, Muse cells, clusters generated from Muse cells (M-clusters), and iPSCs. The results showed that the telomerase activity of Muse cells and M-clusters is much lower than that of iPSCs [33]. In addition, Ogura et al. reported that telomerase activity is similar between adipose-derived MSCs (AD-MSCs) and AD-MSC-derived Muse cells [30]. These results indicate the limited replication and nontumorigenic properties of Muse cells. Furthermore, accumulating evidence has proven that Muse cells do not generate teratomas for at least 6 months, while ESCs and iPSCs form teratomas within 2–3 months after transplantation into immunodeficient mice [20, 30, 31]. Most importantly, to date, nearly 450 MSC clinical trial studies (ClinicalTrials.gov) have been completed worldwide. There have been no cases of tumorigenesis after MSC administration. Thus, Muse cells isolated from various MSC populations should also be safe for clinical application.

### Adhesion-suspension transition

In contrast to MSCs, Muse cells can survive and proliferate in both adherent and suspension states [25]. More importantly, the pluripotency of Muse cells is regulated by an “adherent-suspension switch” [25]. Oct3/4, Sox2, and Nanog are embryonic stem cell markers and have been confirmed as core nuclear transcription factors maintaining cell pluripotency [35]. When adherent cultured Muse cells are transferred to suspension, the expression of *Oct3/4*, *Nanog*, and *Sox2* markedly increases 50 to several hundred times higher than that in adherent culture [35]. Notably, moving Muse cells from a suspension to an adherent state returns the expression of pluripotent genes to their original levels [35]. This indicates that the expression level of these pluripotent genes is reversible between adherent and suspension states. In addition, the suspension state changes the epigenetics of Muse cells [36]. The promoter regions of *Nanog*, *Oct3/4*, and *Sox2* are less methylated in suspension-cultured Muse cells than in adherent-cultured Muse cells [36]. Treating suspension-state Muse cells with a DNA methylation inhibitor upregulates the expression levels of pluripotent genes [36]. However, it is not clear how the suspension state of Muse cells is related to the pluripotent gene methylation state.

Muse cells are in an adherent state when hidden in the body and remain quiescent. Although quiescent Muse cells express Oct3/4, Sox2, and Nanog, these markers are located in the cytoplasm and cannot execute their functions. Once Muse cells detach from their original location and circulate in the peripheral blood, these pluripotent factors enter the nucleus, and their expression is markedly upregulated [25, 35]. Muse cells then acquire pluripotent ability and can repair tissue [25, 35]. The molecular mechanisms by which the adhesion-suspension switch controls the location and expression of pluripotent genes require further investigation [25, 35]. According to immunohistochemical data, Muse cells sporadically reside in connective tissues such as the umbilical cord, adipose tissue, skin, spleen, pancreas, and trachea [31, 32]. Here, these cells exist as round clusters similar to Muse cells in the suspension state in vitro, potentially explaining how they maintain their pluripotent ability [31, 32].

### Stress tolerance

Muse cells secrete serine protease inhibitors (serpins) and 14-3-3 proteins when active [37]. Serpins are an expanding superfamily of structurally similar but functionally diverse proteins [37]. They are the most broadly distributed superfamily of protease inhibitors and have been reported to inhibit caspases and trypsin [38]. Accumulating evidence indicates that 14-3-3 proteins play particularly important roles in G1/S and G2/M cell cycle checkpoint activation, maintenance, and release [39]. If cell damage occurs, 14-3-3 proteins block mitotic entry by regulating cell cycle-related protein kinases and phosphatases [39]. Additionally, 14-3-3 proteins have been reported to protect against stress-induced apoptosis partly by preventing apoptosis and assisting damaged proteins in maintaining their physiologically relevant structure [40]. Therefore, the release of serpins and 14-3-3 proteins may partly account for the stress tolerance of Muse cells. Nicola et al. reported that Muse cells had better resistance to genotoxic stresses than MSCs and non-Muse cells due to their quick and efficient detection of DNA damage and activation of the DNA damage repair system [41]. Muse cells have a more powerful nonhomologous end-joining (NHEJ) DNA repair system than non-Muse cells and MSCs, while there is no significant difference in single-strand repair activity, such as basic excision repair and nucleotide excision repair, among Muse cells, MSCs, and non-Muse cells [21].

### Proliferation of Muse cells

**Fig. 1:**
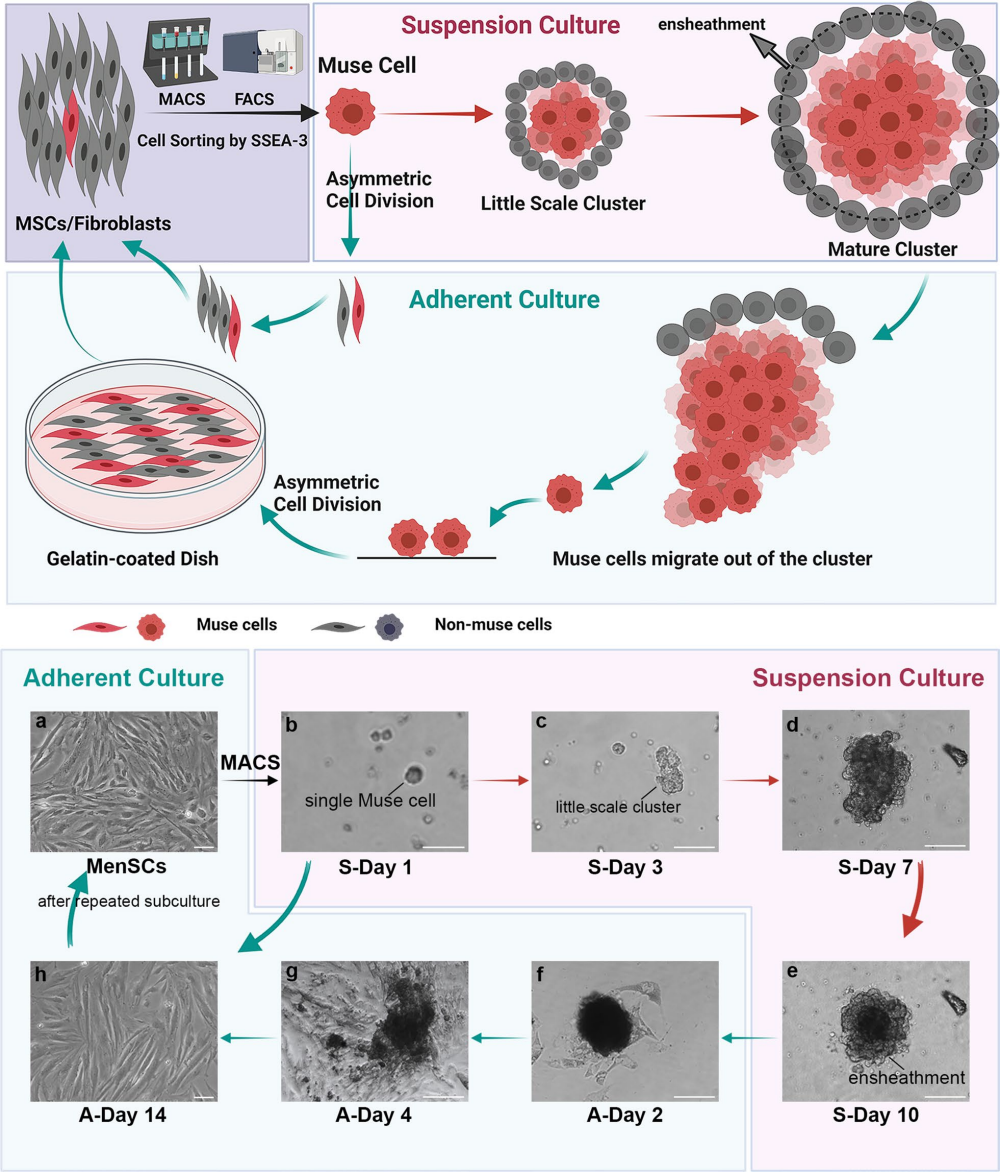
Schematic diagram of the growth process of Muse cells. Muse cells could be isolated from MSC/fibroblast populations by MACS or FACS. In the suspension state, single Muse cells proliferate by asymmetric division and generate both Muse and non-Muse cells. The non-Muse cells enwrap Muse cells, and the enwrapped Muse cells grow gradually from a small-scale cluster to a 50–150 μm mature cluster in 10 days. When the mature cluster is transferred to adherent culture, inner Muse cells migrate out of the cluster and proliferate by asymmetric division. After repeated subculture, the proportion of non-Muse cells accounts for a proportion of cells consistent with that found in MSC/fibroblast populations. Abbreviations: A-Day 2/4/14, adherent culture for 2/4/14 days; FACS, fluorescence-activated cell sorting; MACS, magnetic-activated cell sorting; MenSCs, menstrual blood-derived endometrial stem cells; MSCs, mesenchymal stem cells; S-Day 1/3/7/10, suspension culture for 1/3/7/10 day. Scale bar: 100 μm in **a** and c-h; 50 μm in **b**.

Muse cells proliferate by symmetric and asymmetric cell division manners [20] (Fig. 1). Ninety percent Muse cells can be obtained from MSCs or fibroblasts by fluorescence-activated cell sorting (FACS) or magnetic-activated cell sorting (MACS) [25]. In an adherent culture state, these Muse cells proliferate in an asymmetric manner, which means that one Muse cell will generate one non-Muse cell and one new Muse cell [20]. Therefore, the number of Muse cells gradually decreases and ultimately accounts for a percentage of the total cell population, consistent with the proportion of Muse cells in MSCs and fibroblasts [20]. Shohei Wakao et al. reported that Muse cells in bone marrow and dermis might be the origin of BM-MSCs and fibroblasts in primary culture [42]. In the suspension culture state, single Muse cell generates several slender, flat non-Muse cells at the very early stage of proliferation by asymmetric division, which enwrap Muse cells and gradually generate an ensheathment [25] (Fig. 1b-e). Enwrapped Muse cells proliferate by symmetric division and finally form a 50–150 μm mature cluster within two weeks [20] (Fig. 1e). The morphology, expression pattern of pluripotent markers, and three-germ-layer differentiation potential of the clusters are similar to those of ESCs-derived embryoid bodies [30]. The difference between them is that the outer layer of the M-cluster is always covered by non-Muse cells, while the ESC-derived cluster has no cover cells [25]. In addition, ESCs have unlimited proliferative ability in the suspension culture state, while the growth of M-clusters stops within 2 weeks. The reason for this may be the presence of covering cells or signal changes caused by the suspension state [25]. Interestingly, we found that if the mature M-cluster was not transferred to an adherent culture system in a timely manner, the surrounding non-Muse cells would gradually break away from the M-cluster. Meanwhile, the viability of the Muse cells decreased (data not published). The mechanism by which non-Muse cells affect the growth of the M-cluster remains unknown. When mature M-clusters are transferred to gelatin-coated adherent culture dishes, Muse cells gradually migrate out of the cluster and proliferate in an asymmetric manner until they reach confluence [20] (Fig. 1f-h). After repeated subculture, the proportion of Muse cells was similar to that of MenSCs (Fig. 1h, a). A schematic diagram of the Muse cell growth process is shown in Fig. 1.

### Differentiation potential

Although mesodermal lineage-originated MSCs can cross lineage boundaries and differentiate into ectodermal and endodermal cells, such as neuronal cells [43], insulin-producing cells [44] and hepatocytes [45], under the induction of special factors in vitro*,* the rate of differentiation is generally low, and the induction process is complex. Consistent with the in vitro differentiation results, transplanted MSCs have been reported to ameliorate diseases mainly by secreting various trophic factors and cytokines functioning in immunomodulation, anti-apoptosis, promoting angiogenesis and other mechanisms [46–48]. Few naïve MSCs can spontaneously differentiate into reparative cells in vivo [49, 50]. These phenomena indicate that MSCs are not a homogenous cell population and contain a small subpopulation responsible for their pluripotency. Mari Dezawa’s report in 2010 led to the understanding that this small population of pluripotent stem cells comprises Muse cells. When cultured in suspension, single Muse cell can generate a cell cluster similar to ES cell-derived embryoid bodies. The cluster spontaneously expresses pluripotent markers and exhibits three-germ-layer differentiation potential [20]. Furthermore, these characteristics are retained for up to five generations, indicating that the three-germ-layer differentiation ability of single Muse cell is self-renewable [20, 30]. In contrast, non-Muse cells cannot survive or proliferate in a single suspension culture system [20].

Although it is well known that numerous cell types can be reprogrammed into iPSCs by the four Yamanaka factors, the mechanisms of iPSC generation are controversial [51–54]. There are stochastic and elite model theories [51–54]. Researchers supporting stochastic models believe that while most cells are reprogrammed, only a few successfully become iPSCs. Those supporting the elite models believe that only a specific subpopulation of cells undergoes the reprogramming process and then partially or completely becomes iPSCs [51, 53]. Although the stochastic model is widely accepted, Muse cells provide evidence for the elite models [52]. When fibroblasts were separated into Muse and non-Muse cells and treated with the four Yamanaka factors, both populations could generate clusters. However, only clusters formed by Muse cells had an ES cell-like morphology and expressed the pluripotent genes *Tra-1-81*, *Sox2* and *Nanog *[52]. Only colonies of Muse cells formed iPSCs (Muse-iPSCs) and showed tumorigenic ability after injection into immunodeficient mice [33]. Indeed, the profile of genes related to pluripotency in naïve Muse cells was similar to that in Muse-iPSCs, while naïve non-Muse cells did not express pluripotent genes. Even after induction by Yamanaka factors, the gene expression pattern and level did not change substantially in non-Muse cells [52]. This evidence demonstrates that only a specific population of fibroblast cells can be reprogrammed either partially or completely and that the completely reprogrammed cells ultimately become iPSCs, consistent with the elite model theory [51, 54].

So far, Muse cells have been used in neurological disorders [55–58], acute myocardial infarction [59], adriamycin nephropathy [60], diabetic skin ulcers [61], and liver fibrosis [35]. In these disease models, Muse cells could migrate to lesion sites and spontaneously differentiate into tissue-compatible cells, such as neurons (ectoderm) [6, 12, 55–58, 62], cardiac cells (mesoderm) [59], glomerular cells (mesoderm) [60], vascular endothelial cells (mesoderm) [61] and hepatocytes (entoderm) [35]. Actually, although the differentiation rate is generally very low, Muse cells have spontaneous triploblastic differentiation ability in vitro [26, 62]. In the normal in vitro culture, Muse cells were positive for Nestin (~ 1.9%, ectoderm), MAP-2 (~ 3.8%, ectoderm), *α*-SMA (8.0 ± 0.6%, mesoderm), and α-fetoprotein (3.2 ± 0.3%, entoderm) [56, 63]. After transplantation into lesion sites, the differentiation rate of Muse cells could have a greatly increase. In a subacute lacunar stroke model, about 62% grafted naïve Muse cells spontaneously differentiated into neurons indicated by expressing NeuN. Meanwhile, about 12% grafted cells expressed oligodendrocyte marker GST-pi. Interestingly, the grafted Muse cells did not express microglia or astrocyte markers. Further evidence showed the injured region was mainly composed of neuronal cells and oligodendrocytes, with a small fraction of microglia and astrocyte. These results suggested the microenvironmental cues in the lesion site may play a vital role in the fate determination of grafted Muse cells [56]. We speculate that after homing to the lesion site, Muse cells may be affected by the specific microenvironment, which consists of multifarious cytokines, growth factors, chemokines, and extracellular matrix components. Then, upon interacting with the local factors, the lineage commitment of Muse cells will be initiated or restrained. Moreover, the initiated cellular and molecular signalling pathways interact with one another, forming a sophisticated regulatory network. As a consequence of the delicate balance, Muse cells eventually differentiate in a particular direction.

Although the complex mechanisms and pathways by which Muse cells could differentiate into tissue-compatible cells are far from fully understood, transcription factors and signalling pathways that affect the differentiation of MSCs are gradually being elucidated especially in the process of adipogenesis, osteogenesis, chondrogenesis, and myogenic [64–66]. Signalling of cyclic AMP, neurotrophin, wingless-type MMTV integration site (Wnt), retinoic acid, and bone morphogenic protein (BMP), etc. have been reported to regulate the differentiation of MSCs to neuroglia [67]. Besides, Xie et al. and Yang et al. reported circular RNAs were involved in the differentiation of NSCs in rat and mouse brain [68, 69]. It needs to be further studied which signalling pathways or transcription factors control the differentiation of Muse cells into a specific direction. Thus, with delivering of related genes into Muse cells, the differentiation rate to an intended cell lineage may get improved after transplantation to the lesion site. Accordingly, Muse cells can achieve better therapeutic effect in various diseases.

### The preferential homing mechanisms of Muse cells

The correlation between Muse cells in the peripheral blood and tissue damage has been reported by several clinical studies. Among 29 recruited ischaemic stroke patients, 27.6% showed a robust increase of peripheral blood-Muse cells within 24 h after stroke onset, suggesting that Muse cells could be mobilized from the bone marrow into peripheral blood at the acute stage of ischaemic stroke [70]. A similar phenomenon was observed in acute myocardial infarction patients [27]. In an in vitro coculture system, Muse and non-Muse cells in the upper chamber both had the potential to migrate to slices of damaged tissues or serum from disease models placed in the lower chamber. However, the number of migrated Muse cells was significantly higher than that of non-Muse cells, consistent with the findings of the in vivo studies [35, 60].

When MSCs are intravenously injected into recipients, the majority become trapped in the lung capillaries [71]. However, most Muse cells can escape from the entrapment of lung capillaries [72]. Given that the sizes of Muse and non-Muse cells are not notably different, the mechanism by which Muse cells travel through the lung capillaries may be unrelated to cell size. The stromal-derived factor 1 (SDF-1)-CXCR4 axis is known to mediate the migration and homing of MSCs to damaged tissues [41]. Muse and non-Muse cells express the same level of *CXCR4*. An antagonist of CXCR4 partially suppresses the migration of Muse cells but fails to completely abrogate this process [35]. Therefore, the SDF-CXCR4 system cannot fully explain the preferential migration of Muse cells to damaged tissue. SIP is a signal for acute inflammation/damage and is produced by damaged cells [73]. Among the five *SIPR* subtypes (*SIPR1*-*SIPR5*), the expression level of *SIPR2* in Muse cells was significantly higher than that in non-Muse cells, while the expression level of the other *SIPR* subtypes was lower than that in non-Muse cells [74]. Accumulating evidence has demonstrated that SIP and its receptor SIPR2 may play a central role in controlling the preferential migration of Muse cells both in vitro and in vivo. Yamada et al. showed that an antagonist of SIPR2 could inhibit the migration of Muse cells to postinfarct heart slices in vitro and to myocardial infarction tissue in vivo. Consistently, the application of an antagonist of SIPR2 attenuated the therapeutic effect of Muse cells in an acute myocardial infarction model [74]. In acute myocardial infarction patients, the level of serum SIP was significantly elevated after disease onset (day 0) and peaked on day 1. By comparison, the number of Muse cells in peripheral blood did not show an obvious change on day 0 but increased on day 1 and then gradually returned to baseline in the following several weeks [74]. These findings indicate that the SIP-SIPR2 axis greatly contributes to the efficient homing of Muse cells to damage sites.

## Comprehensive comparison of Muse cells and other stem cells

### Muse cells *versus* non-Muse cells

As mentioned above, Muse cells are a subpopulation of MSCs that can be distinguished from other cells by SSEA-3. Thus, Muse cells are positive for both pluripotent and mesenchymal markers, while non-Muse MSCs are only positive for mesenchymal markers [75]. Muse cells show better resistance to physical and chemical genotoxic stress than non-Muse cells [21]. Although the single-strand repair system efficiency is the same in these two populations of cells, the double-strand repair system (nonhomologous end-joining) in Muse cells is more powerful than that in non-Muse cells [21]. Muse cells express pluripotency-related genes such as *Oct3/4*, *Sox2*, *Nanog*, *Rex1*, and *PAR4*, while non-Muse cells do not [32]. Moreover, individual Muse cells can form ESCs-like clusters in suspension and exhibit three-germ-layer differentiation potential, while single non-Muse cell cannot survive in suspension [20]. Muse cells are able to differentiate into mesodermal lineage cells (skeletal muscle, cardiomyocytes [36], glomerular cells), endodermal lineage cells (hepatocytes [35], cholangiocytes), and ectodermal lineage cells (melanocytes [76], neuronal cells [63], keratinocytes [77]). By comparison, the differentiation ability of non-Muse MSCs is limited to adipocytes, osteocytes, and chondrocytes, and their differentiated proportion is lower than that of Muse cells. When fibroblasts are separated into Muse cells and non-Muse cells and transfected with 4 Yamanaka genes, only colonies formed by Muse cells exhibit ESC embryoid body-like morphology and express TRA-1–81, a marker for identifying promising iPS colonies [33]. Muse cells excluding fibroblasts cannot generate iPSC colonies. The induction efficiency of iPSCs by naïve human skin fibroblasts is approximately 0.001%, while fibroblast-derived Muse cells generate iPSCs 30 times more efficiently than naïve fibroblasts [33]. These differences between Muse and non-Muse cells are listed in Table 1.

**Table 1:** Differences between Muse and non-Muse cells

	Muse cells	Non-Muse MSCs/Fibroblast
SSEA3 expression	+	–
Pluripotent related genes expression	+	–
Trilineage differentiation	+	–
ESCs- like clusters	+	–
iPSCs generation ability	+	–
Growth character	Suspension/Adhesion	Only adhesion
Division method	Symmetric /Asymmetric division	Symmetric division
Stress tolerance	+ + +	+
Homing ability	+	–
Spontaneous differentiation in vivo	+	–

### Muse cells *versus *ESCs *versus* iPSCs

**Fig. 2:**
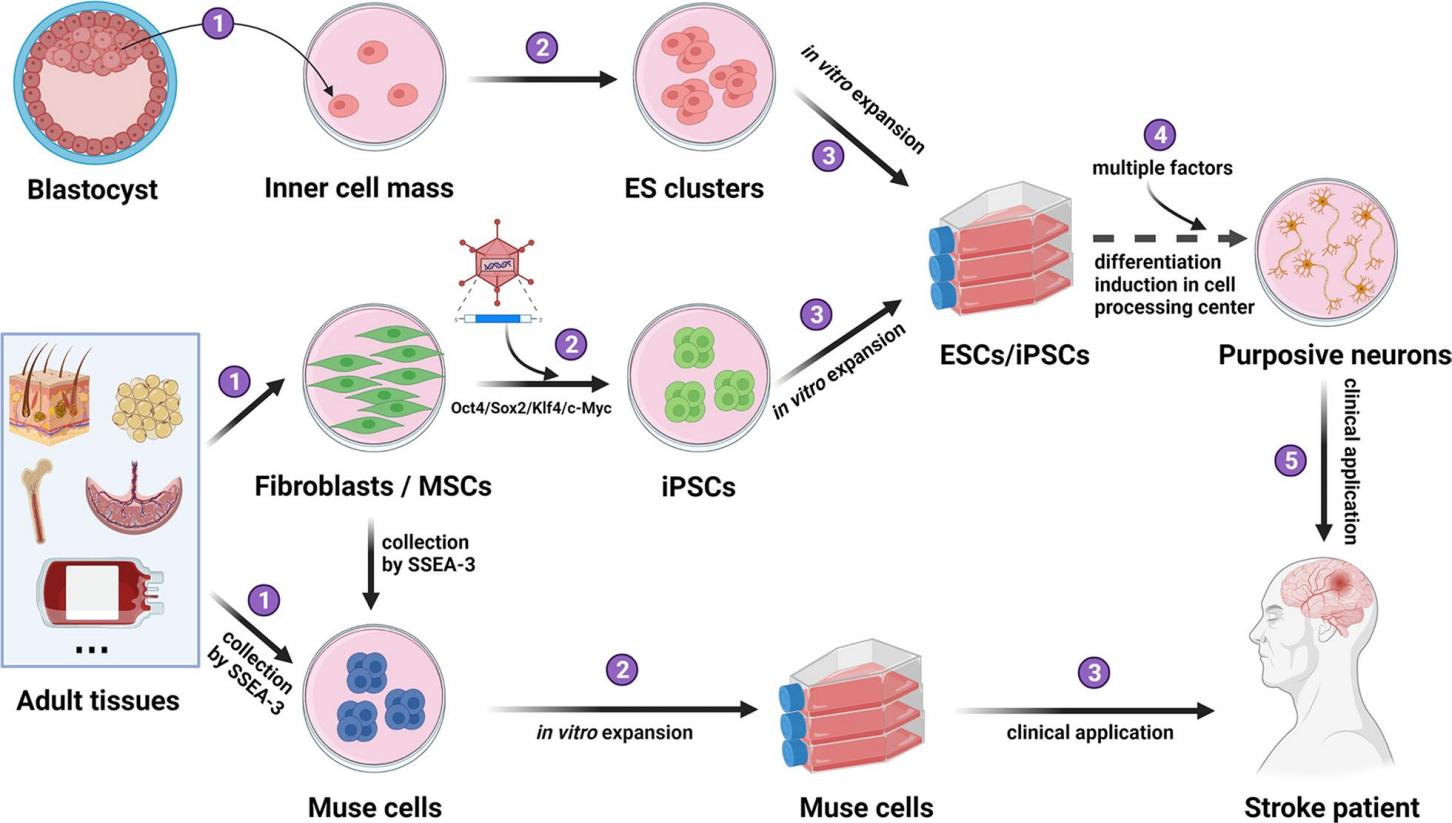
Necessary steps for the clinical application of ESCs, iPSCs, and Muse cells. Five steps are needed for the clinical application of ESCs and iPSCs, while only three simple steps are needed for the use of Muse cells. Abbreviations: ESCs, embryonic stem cells; iPSCs, induced pluripotent stem cells.

The differences among ESCs, iPSCs, MSCs and Muse cells are listed in Table 2. Briefly, the availability of ESCs is very limited, these cells are associated with serious ethical concerns, and autologous transplantation cannot be performed. Muse cells are more readily accessible and are associated with fewer ethical concerns. Muse cells can be obtained by FACS or MACS with the specific surface marker SSEA-3 without any induction of factors or gene manipulation [26]. In addition, after simple expansion in vitro, Muse cells can be directly transplanted into animals or humans. In summary, only three simple steps are needed for the use of Muse cells: collection by SSEA-3; expansion in vitro; and injection into patients. In contrast, ESCs and iPSCs must be induced before transplantation to prevent tumorigenesis (Fig. 2) [25]. This process is complicated, time-consuming, mechanism unclear, and expensive because of the introduction of various factors [32]. Additionally, the multiple induction steps complicate the quality control process and affect consistency among product batches [32]. These characteristics limit the clinical use of ESCs and iPSCs. Due to their excellent differentiation ability, ESCs and iPSCs exert their reparative effect mainly by generating new tissue-specific cells and facilitating tissue regeneration, while MSCs improve disease mainly through their robust paracrine effects [46–48]. Muse cells have been reported to play a therapeutic role through immunoregulation, antifibrotic, and antiapoptotic mechanisms, as well as cellular replacement by spontaneously differentiating into specific cells [25]. Human leukocyte antigen-G (HLA-G) is an immune tolerance factor expressed in immune-privileged organs such as the placenta, thymus, ovaries and testes [78]. The expression rate of HLA-G in Muse cells is approximately 90%, significantly higher than that in other types of stem cells [25]. For example, human ESCs and iPSCs do not express *HLA-G *[79], and the expression rate of *HLA-G* in adult BM-MSCs is less than 20% [80]. The high expression of HLA-G and the immunomodulatory effect of Muse cells may help them escape immune attack in the early stage of integration into tissue, facilitating tissue reconstruction. The most advantageous property of Muse cells is their stress-enduring ability, allowing them to survive for a prolonged period in damaged sites after transplantation. Thus, Muse cells are promising candidates for disease treatment.

**Table 2:** Comprehensive comparison of Muse cells and other stem cells

	ESCs	iPSCs	MSCs	Muse cells
Origin	Embryos	Somatic cells	Various tissues	Various tissues
Richness	–	+	+	–
Ethical problem	+ + +	-	-	–
Type	Pluripotent	Pluripotent	Multipotent	Pluripotent
Three-germ layers differentiation	+ + +	+ + +	+	+ + +
Immunogenicity	+	+	–	–
Autologous	–	+	+	+
Immunomodulation	–	–	+	+
Tumorigenicity	+	+	–	–
HLA matching	+	–	–	–
Technological difficulty of isolation/culture	+	+	–	–
Grow character	Suspension/adhesion	Adhesion	Adhesion	Suspension/adhesion
Division method	Symmetric	Symmetric	Symmetric	Symmetric /asymmetric
Stress tolerance	+	+	+	+ + +
Differentiation prior to transplantation	Necessary	Necessary	Not necessary	Not necessary

### Pre-clinical studies of Muse cells on stroke

The lack of neuronal regenerative ability causes disability and death in many nervous system diseases, including stroke. Muse cells can be induced or spontaneously differentiate into neural stem cells or neural lineage cells in vitro [30, 33, 63]. Cells generated from a single Muse cluster can be differentiated into neural spheres expressing neural stem cell markers, such as Nestin, Musashi, and NeuroD. With further neural induction, approximately 89% of cells are positive for MAP-2 or GFAP [33]. Additionally, even without neural differentiation induction, Muse cells can spontaneously differentiate into cells positive for neural stem cell (~ 1.9% Nestin), mature neural (~ 3.8% MAP-2), astrocyte (~ 3.4% GFAP) and oligocyte (~ 2.9% O4) markers [63]. The in vitro neural differentiation ability of Muse cells was also proven in Ogura’s study [30]. They reported that AD-MSC-derived Muse cells can be differentiated into Tuj-1-positive cells in vitro, while non-Muse cells cannot [30]. These in vitro studies provide a theoretical basis for the application of Muse cells for treating central nervous system diseases.

Several pre-clinical studies compared the therapeutic effect of Muse cells, non-Muse cells or their parental MSCs on stroke. In a severe combined immunodeficiency (SCID) mouse ischaemic model constructed by permanent middle cerebral artery occlusion (pMCAO), vehicle, BM-MSCs, Muse cells (SSEA3^+^ BM-MSCs), and non-Muse cells (SSEA3^-^ BM-MSCs) were transplanted into the ipsilateral striatum at 7 days after the onset of pMCAO [81]. The transplantation of BM-MSCs and non-Muse cells significantly improved animal motor function, as assessed by rotarod treadmill activity, at 21 days after transplantation. The effect plateaued at 28 days. In contrast, the therapeutic effect of Muse cells was obviously observed at 35 days post-transplantation, which was 2 weeks later than that in the BM-MSCs and non-Muse cells groups. Anti-human mitochondrial antibody (hMit) was used to detect the transplanted cells at 42 days post-transplantation. The results showed that numerous Muse cells migrated and integrated into the peri-infarct cortex, while the number of integrated human BM-MSCs or non-Muse cells was negligible. Furthermore, Muse cells spontaneously differentiated into Tuj-1 (~ 45.3%) and NeuN-expressing cells (~ 20.5%), and very few differentiated into GFAP-positive cells (~ 1.4%). These findings indicated that Muse cells might contribute to tissue regeneration and functional recovery by homing to the tissue and differentiating into tissue-specific cells, while non-Muse cells mainly contribute through paracrine effects [81].

In a rat transient middle cerebral artery occlusion (tMCAO) model, Muse cells derived from normal human dermal fibroblasts (NHDFs) were proven to facilitate functional recovery, as evaluated by the modified neurologic severity score (mNSS) and rotarod test at 70 and 84 days after transplantation [63]. In sharp contrast, functional recovery was not observed in the non-Muse transplantation group or vehicle group at any time point [63]. hMit immunofluorescence results showed that a large number of Muse cells could survive in the peri-infarct area for up to 84 days, while non-Muse cells were barely detectable [63]. Furthermore, the surviving Muse cells were positive for the mature neuron markers NeuN (~ 64.6%) and MAP-2 (~ 32.4%), interneuron marker calbindin (~ 27.5%), and oligodendrocyte marker GST-π (~ 25%) [63]. Although the proportion was small, Muse cells were shown to spontaneously differentiate into neural lineage cells in in vitro culture and were positive for neurofilaments (~ 2.6%) [56], Nestin (~ 1.9%), MAP-2 (~ 3.8%), GFAP (~ 3.4%), and oligodendrocyte marker O4 (~ 2.9%) [63]. However, non-Muse cells did not express any of the above neural lineage markers [63]. These findings indicate that Muse cells may be affected by the damaged microenvironment of the host brain and be induced to differentiate into neural lineage cells to replenish lost neurons [63]. A retrograde tracing experiment showed that Muse cells could integrate into the sensory-motor cortex, extend neurites into the pyramidal tract, and display normalized hindlimb somatosensory evoked potentials [63].

In contrast to MCAO, lacunar infarction induces small subcortical infarction, accounting for approximately 25% of all types of ischaemic stroke. In a lacunar infarction model of SCID mice, 1 × 10^5^ human BM-MSCs or BM-MSC-derived Muse cells were stereotaxically injected into the perilesion site [56]. Eight weeks after transplantation, ~ 28% of initially transplanted Muse cells integrated into the host brain, and most of them were distributed around the lesion site [56]. In contrast, few MSCs were detected in the brain at the same time point [56]. These integrated Muse cells were positive for NeuN (62.2 ± 2.4%), MAP2 (30.6 ± 3.1%), and GST-π (12.1 ± 1.1%) but negative for GFAP, Iba-1, and Ki67 [56]. These results suggested that the surviving Muse cells did not proliferate in vivo and tended to differentiate into neurons instead of into glial cells. Anterograde dextran tracing showed that Muse cells facilitated pyramidal tract reconstruction [56]. The corner turn test and cylinder test demonstrated significant behaviour improvement in the Muse group compared with the MSC and vehicle groups at 6 and 8 weeks post-transplantation [56]. Moreover, the safety of Muse cells was proven by screening for tumour formation in different organs (the brain, lung, kidney, liver, and spleen) at 6 months after transplantation [56].

Abe et al. studied the appropriate transplantation time and dose of Muse cells by cervical vein injection in an immunodeficient mouse lacunar model [82]. Different doses of CL2020 (product name of Muse cells), 5 × 10^4^ cells (high-dose group), 1 × 10^4^ cells (medium-dose group), and 5 × 10^3^ cells (low-dose group), were administered to the SCID mouse lacunar model at the subacute phase (~ 9 days after onset) and chronic phase (~ 30 days after onset) [82]. CL2020 was mainly distributed in the peri-infarct area at 22 weeks post-transplantation and expressed NeuN and MAP-2 [82]. The high-dose group showed obvious functional recovery compared with that of the vehicle group in both subacute-phase and chronic-phase animals, and this effect persisted for up to 22 weeks without tumorigenesis and adverse effects [82]. Furthermore, a loss-of-function study demonstrated that intraperitoneal injection of diphtheria toxin could abrogate the functional recovery mediated by CL2020 [82].

**Fig. 3:**
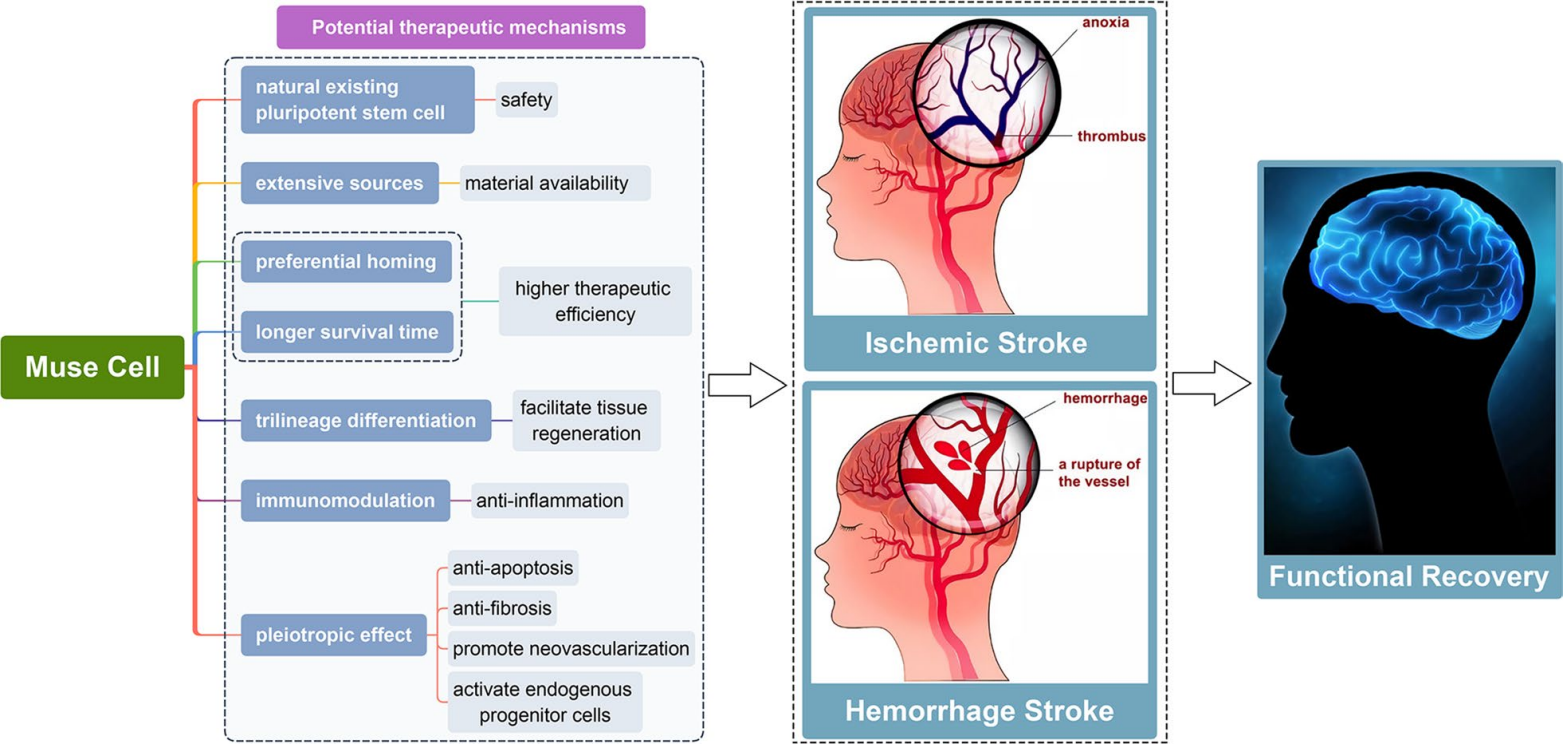
Potential therapeutic mechanisms of Muse cells in stroke. Muse cells are naturally existing pluripotent stem cells for which there are numerous sources. Muse cells may improve ischaemic and haemorrhagic stroke through preferential homing, longer survival time in hostile environments, three-germ-layer differentiation capacity, immunomodulation capacity and pleiotropic effects

Norihito et al. studied the efficiency of Muse cells in a mouse intracerebral haemorrhage (ICH) model [83]. Five days after ICH onset, 2 × 10^5^ Muse cells isolated from BM-MSCs, non-Muse cells or PBS were injected into the ICH cavity [83]. The motor function recovery of mice in the Muse group was superior to those in the non-Muse and PBS groups five days post-transplantation and was maintained at day 68 [83]. In the non-Muse group, functional recovery became significant at day 54 compared with that in the vehicle group but was not as notable as that in the Muse group [83]. Histological results showed that the number of Muse cells in the host brain was approximately 9 times greater than that of non-Muse cells at 69 days, and they differentiated into NeuN (~ 57%) and MAP-2 (~ 57%)-positive neurons in the host brain [83]. The pre-clinical studies evaluating Muse cells in stroke are listed in Table 3, and the potential mechanisms of Muse cells in stroke are described in Fig. 3.

**Table 3:** Pre-clinical studies of Muse cells on stroke

Animal	Model type	Cell type	Transplantation time/method/region/ dose	Main results	Reference
SCID mouse	pMCAO	Human BM-MSCs derived Muse cells	7 days after injury onset; stereotaxical injection to ipsilateral striatum; 2.5 × 10^4^	Migrate and survive in the damaged site; differentiate to neurons; function recovery and tissue regeneration	[77]
Rat	tMCAO	NHDFs-derived Muse cells	2 days after injury onset; stereotaxical injection to ischaemic cortex; 3 × 10^4^	Survive up to 84 days; integrate into sensory-motor cortex; differentiate to neurons in cortex	[60]
SCID mouse	lacunar infarction	Human BM-MSCs derived Muse cells	2 weeks after injury onset; stereotaxical injection to perilesion site; 1 × 10^5^	Integrate into host brain; facilitate pyramidal tract reconstruction; differentiate to neurons; improve behaviour score; without tumour formation by 6 months	[78]
SCID mouse	lacunar infarction	CL2020	9 days or 30 days after injury onset; cervical vein injection; 5 × 10^4^/1 × 10^4^/5 × 10^3^	Survive in host brain for at least 22 weeks; without tumorigenesis; differentiate to neurons; behaviour improvement	[79]
SCID mouse	ICH	Human BM-MSCs derived Muse cells	5 days after injury onset; stereotaxical injection to hematoma cavity; 2 × 10^5^	Improve motor function recovery; differentiate to neurons; much higher survival rate of Muse cells compared with non-Muse cells	[80]

### Obstacles and challenges of Muse cells from bench to bedside

Due to the properties of nontumorigenic, effective homing, stress tolerance, and differentiation to tissue compatibility cells after transplantation, Muse cells have become a promising cell source for disease treatment. Nevertheless, some noticeable obstacles need to overcome before Muse cells can be responsibly transferred to novel therapies.

The most important thing is to establish a standard criterion for the sorting methods of Muse cells. Fluorescence-activated cell sorting (FACS) [26, 55, 63], magnetic-activated cell sorting (MACS) [28, 61, 84], and long-term trypsin incubation (LTT) [85–87] are three commonly used methods to achieve Muse cells. Uchida et al. [56] suggested Muse cells isolated by MACS had higher cell viability and the collection efficiency was better compared with FACS. Nevertheless, the purity of Muse cells sorted by magnetic beads was lower than flow cytometry. No application of SSEA-3 makes LTT the most economical method compared with the other two, while the purity of Muse cells is the most unsatisfied. Therefore, some researchers term the cells isolated by LTT as Muse-enriched cell (MEC) population. Additionally, Kuroda et al. mentioned different brand SSEA-3 antibody and culture procedures of parent cells including thawing, growing, passaging, freezing, and serum choice would greatly affect the yield of Muse cells [26]. Secondly, bone marrow, adipose, various MSCs, and fibroblast are common sources for Muse cells. Whether the variability of sources, donor age, and passage of parent cells affect the gene/protein expression profiles and the therapeutic outcome of Muse cells are still unknown. Golden standardization is urgently needed to produce high quality and high consistency Muse cells.

Thirdly, since Muse cells only occupy a small population in various sources, it takes time to expand to adequate cells for clinical administration. Besides, the narrow time window of stroke makes it impossible for patients to use freshly prepared Muse cells. Thus, autologous or allogeneic cryobanked Muse cells will be the main force of clinical application. Cryopreservation companies should include the storage of Muse cells in their business scope, and establish strict GMP-compliant procedures to provide high quality cells to patients.

Lastly, because adherent Muse cells proliferate in an asymmetric manner, the proportion of Muse cells is bound to gradually decrease following repeated passaging. Then, another cycle of sorting will be applied to enrich Muse cells. Therefore, compared with MSCs, the culture cost of Muse cells is more expensive and the culture procedures are more complicated. Complex steps will introduce more variable parameters, resulting in inconsistent quality between different batches of cell products, which ultimately lead to widely varying treatment outcomes after clinical transplantation.

## Conclusion

Despite the potential challenges remain to be solved, Muse cells could compensate the shortcomings of MSCs, iPSCs and ESCs, and represent an important therapeutic tool to improve the outcome of stroke. Since the first reported in 2010, studies about Muse cells have been going on for 12 years. However, compared with MSCs, there are not as many as online published articles and they are still largely unexplored. Hopefully, more researchers could pay attention to Muse cells, dig deeper to elucidate their therapeutic mechanisms and potential, and make this cell source a clinically competitive treatment method in the near future.

## Data Availability

Not applicable.

## Abbreviations

AD-MSCs: Adipose-derived MSCs
BM-MNCs: Bone marrow mononuclear cells
BM-MSCs: Bone marrow-derived MSCs
CXCR4: Chemokine (C-X-C motif) receptor 4
ESCs: Embryonic stem cells
FACS: Fluorescence-activated cell sorting
HLA-G: Human leukocyte antigen-G
hMit: Anti-human mitochondrial antibody
ICH: Intracerebral haemorrhage
iPSCs: Induced pluripotent stem cell
MACS: Magnetic-activated cell sorting
M-clusters: Clusters generated from Muse cells
MenSCs: Menstrual blood-derived endometrial stem cells
MSCs: Mesenchymal stem cells
Muse: Multilineage differentiating stress enduring
NHEJ: Nonhomologous end-joining
NSCs/NPCs: Neural stem/progenitor cells
pMCAO: Permanent middle cerebral artery occlusion
SCID: Severe combined immunodeficiency
SDF-1: Stromal-derived factor 1
serpins: Serine protease inhibitors
SSEA-3: Stage-specific embryonic antigen 3
tMCAO: Transient middle cerebral artery occlusion
TPA: Tissue-type plasminogen activator
UC-MSCs: Umbilical cord-derived MSCs
